# Severe Short Stature: an unusual finding in lipoid proteinosis

**DOI:** 10.4008/jcrpe.v1i2.31

**Published:** 2008-11-06

**Authors:** Şükran Poyrazoğlu, Hülya Günöz, Feyza Darendeliler

**Affiliations:** 1 İstanbul University, İstanbul Faculty of Medicine, Department of Pediatrics, Pediatric Endocrinology Unit, İstanbul, Turkey; +90 212 532 42 33+90 212 533 13 83sukranpoyrazoglu@yahoo.comİstanbul University, İstanbul Faculty of Medicine, Department of Pediatrics, Pediatric Endocrinology Unit, İstanbul, Turkey

**Keywords:** short stature, Genodermatosis, lipoid proteinosis

## Abstract

Lipoid proteinosis (LP) is a rare disorder and it can affect every organ in the body. The clinical manifestations of LP may vary considerably between affected individuals. Short stature is reported in patients with LP however the underlying etiology is not clear. Short stature may be due to endocrine dysfunction caused by deposition of hyaline−like material in endocrine glands. We investigated a 13 year old patient with LP (507 delT mutation) who had severe short stature. He had hoarseness since the age of one year, followed by characteristic skin lesions for LP and short stature. There was no pathology with respect to endocrinological investigations in our patient including growth hormone−IGF axis. Our results show that short stature in LP can not be explained by endocrinological abnormalities. Short stature may be an intrinsic component of the syndrome.

**Conflict of interest:**None declared.

## INTRODUCTION

Lipoid proteinosis (LP) is a condition characterized by the deposition of periodic acid Schiff−positive hyaline−like material in various tissues including skin, mucous membranes and internal organs.(1, 2) The etiology of LP is currently unknown. It has been shown that LP results from mutations in extracellular matrix protein 1 (ECM1) genes on chromosome 1q21. Although classical features include laryngeal infiltration leading to hoarseness, scarring and infiltrations of the skin, LP can affect every organ in the body.(1, 2) Recurrent episodes of inflamed parotid and submandibular glands may occur. A shortened tongue with a thickened frenulum may also be seen.(3) Other extracutaneous features include epilepsy and neuropsychiatric abnormalities, sometimes in association with calcifications in the temporal lobes or in the hippocampus.(4, 5) Deposition of hyalinelike material in the eye may cause corneal opacities or secondary glaucoma. Intestinal bleeding may occur as a result of deposition in the small bowel.(6, 7) Lung and bronchial involvement has also been reported in patients with LP.(8) Insulin resistance was also detected.(9) Chakrabarti et al.(10) reported a patient with LP and dwarfism. The cause of dwarfism could not be elicited in this patient and it was postulated that the short stature could have been due to defective osteoblasts which are biologically similar to fibroblasts. Endocrinological dysfunction was not evaluated in this patient. Recently, short stature was reported in two LP patients from two different families.(11) Considering the possibility that short stature in LP patients may be due to endocrine dysfunction caused by deposition of hyaline−like material in endocrine glands, we investigated one of the patients reported below (Patient 1) for endocrinological involvement.

## CASE REPORTS

**Figure 1:**
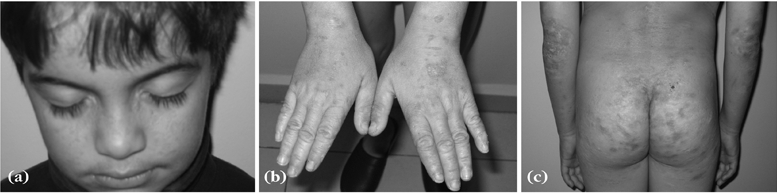
Multiple acneiform scars on face and beaded papules on the margins of eyelids (a), papulovesicular lesions, scars and hyperkeratosis on hands (b), elbows and buttocks (c) of lipoid proteinosis patient with short stature.

Patient 1. A 13−year−old boy had been referred to our clinic with a history of hoarseness since the age of one year, followed by skin lesions and short stature. He was the offspring of a second degree consanguineous marriage. His height was 128.2 cm, weight 25.9 kg and body mass index 15.8. Expressed as standard deviation scores (SDS), these values were –3.9 SDS, –3.1 SDS and –1.6 SDS respectively, according to age and sex specific Turkish standards.(12, 13) Sitting height/ height ratio was normal for age (0.52). Mother’s height was 168.5 cm (0.9 SD) and father’s height was 175.1 cm (–0.2 SD). Physical examination revealed multiple acneiform scars on the face; beaded papules on the margins of the eyelids; papulovesicular lesions, scars and skin hyperkeratosis on the hands, elbows, knees, axilla and buttocks (Figure 1). Scarring was reported following trauma or injury. The cutaneous changes were unrelated to sun exposure. The patient’s tongue was firm, thick, pale, with yellowish−white papules on its surface. The mobility of the tongue was restricted by a thickened frenulum. Dentition was normal. There were no signs of puberty. Developmental milestones were normal.

Routine blood chemistry, lipid profile, complete blood count, urine analysis and stool examination were all within the normal ranges. Bone age corresponded to age 10 years, according to the Greulich and Pyle method.(14) The electroencephalogram was normal. Further investigations were undertaken to detect deposition of hyaline−like material at various sites. Fundoscopic and slit lamp examination of the eyes revealed normal results. Laryngoscopic examination showed mucosal thickening in the vocal cords and subglottic region. Serum levels of vitamin A, zinc and copper were normal.

Hoarseness and characteristic skin changes of the patient was pathognomonic for LP. Histopathological examination of skin biopsy specimen showed orthokeratotic hyperkeratosis and acanthosis of epidermis, hyperkeratosis, acanthosis and evident bunch of collagen arranged in parallel to surface, and tight and fibrotic appearance of dermis and ectasia of some small blood vessels, some hyaline material deposition and hyaline appearance of basal lamina and fibrosis of papillary dermis.

The results of endocrinological investigations are shown in Table 1. Serum levels of thyroid hormone, insulin like growth factor− I (IGF−I) and IGF−binding protein−3 (IGFBP− 3) were normal. Growth hormone (GH) secretion investigated by GH tests and sensitivity investigated by IGF−1 response to GH injection did not reveal GH deficiency or GH insensitivity. Adrenal cortex function was also normal at insulin tolerance test. Gonadotropin− releasing hormone test and testosterone response to human chorionic gonadotropin (HCG) stimulation were within normal ranges for age and pubertal status, indicative of a normally functioning hypothalamo−pituitary−gonadal axis.

Oral glucose tolerance test, performed for evaluation of insulin resistance, revealed normal results. Magnetic scanning showed calcification in both amygdals. Pituitary gland imaging results were unremarkable.

For investigation of osteoblastic activity and bone turnover, bone mineral density of the lumbar L1−L4 vertebrae, serum levels of calcium, phosphorus and bone turnover markers were evaluated. The results were normal (Table 2). Endoscopic examination of the upper gastrointestinal tract and small bowel biopsy also gave normal results.

Patient 2. The family history of Patient 1 revealed that one of his younger cousins (a 6 years old male) also had a hoarse voice and very mild cutaneous findings. On examination, this patient’s height and weight were at the 10^th^ percentile for age. His mental development was normal. His parents were also first cousins.

Informed consent for genetic analysis was obtained from the families of both patients. The genetic analyses were performed in St John’s Institute of Dermatology, London, UK. Both patients were homozygous for mutation 507delT in exon 6 of ECM1. Genetic analysis of the parents yielded a heterogeneous mutation 507delT in both parents of patient 1 and in the father of patient 2.

**Table 1:**
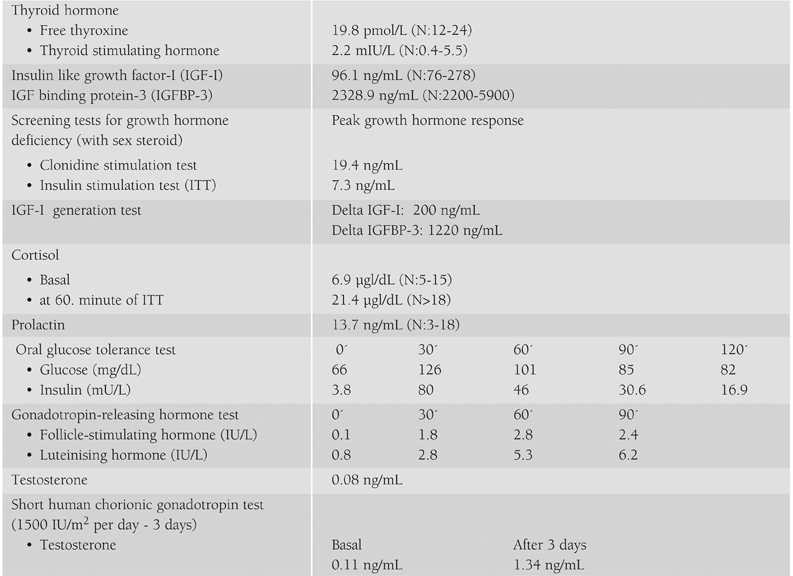
Results of endocrinological investigations in Patient 1.

**Table 2:**
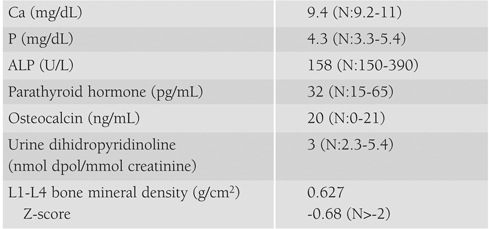
Osteoblastic activity markers, bone turnover markers and bone mineral density in Patient 1.

## DISCUSSION

LP results from mutations in ECM1, a glycoprotein expressed in several tissues including the skin. This glycoprotein is composed of two alternatively spliced isoforms, ECM1a and ECM1b, the latter lacking exon 7 of this 10−exon gene. The mutations map onto chromosome 1q21. To date, mutations that either affect ECM1a alone or that disturb both ECM1 transcripts have been demonstrated in several cases. Over 20 different mutations have been detected throughout the world.(1, 2) Exons 6 and 7 are the most common sites for ECM1 mutations in LP. Clinically, it appears that mutations outside exon 7 are usually associated with a slightly more severe mucocutaneous LP phenotype. Neurological features do not show any specific genotype−phenotype correlation.(1, 2)

The clinical manifestations of LP may vary considerably between affected individuals. Individuals with the same genetic background and the identical mutation were shown to have a vastly different phenotype.(1, 2) Homozygous 507delT mutation was detected in our patients. Although both of our patients have the same mutation, the severity of disease was very different in the two patients. This particular mutation has been documented previously. Two Thai brothers with temporal lobe epilepsy and a 3−year−old Iranian male only with skin lesions and hoarseness were reported.(2) Uchida et al.(15) detected the same mutation in a patient with mucocutaneous lymphangiogenesis.

Chakrabarti et al.(10) reported a patient with LP and dwarfism. The cause of dwarfism could not be elicited in this patient and it was postulated that the short stature may have been be due to defective osteoblasts which are biologically similar to fibroblasts. Our patient also has short stature. However, his osteoblastic activity markers, bone turnover markers and bone mineral density were all normal.

Endocrinological investigations based on the possibility that short stature in LP patients may be due to endocrine dysfunction caused by deposition of hyaline−like material in the endocrine glands also failed to detect any endocrinological organ involvement.

Our results show that short stature in LP cannot be explained by endocrinological abnormalities. We postulate that short stature in LP patients probably constitutes one of the components of this rare syndrome with a hitherto unknown etiology. Further cases with short stature, if any, will enlighten this association.

## ACKNOWLEDGEMENT

Sequencing of the ECM1 gene was kindly performed by Dr Lu Liu and Professor John McGrath, St John’s Institute of Dermatology, London, UK.
